# Time series analysis and scaling law characteristics of viral hepatitis from 2004 to 2023 in Zhejiang Province, China

**DOI:** 10.1371/journal.pone.0319642

**Published:** 2025-03-10

**Authors:** Ming Xue, Chen Wu, Kui Liu, Qinbao Lu, Zheyuan Ding, Xinyi Wang, Tianyin Fu, Junfen Lin, Haocheng Wu

**Affiliations:** 1 Hangzhou Center for Disease Control and Prevention (Hangzhou Health Supervision Institution), Hangzhou, Zhejiang, China; 2 Zhejiang Province Center for Disease Control and Prevention, Hangzhou, Zhejiang, China; 3 Zhejiang Key Lab of Vaccine, Infectious Disease Prevention and Control, Hangzhou, Zhejiang, China; Affiliated Hospital of Nantong University, CHINA

## Abstract

**Background:**

Hepatitis significantly increases the global disease burden and has become a major public health issue worldwide. China is a high-risk area for viral hepatitis, which is also a serious public health problem.

**Methods:**

The scaling relationship between various types of hepatitis and population size was explained by a scaling law. Fixed-effects and random-effects meta-analyses were used to calculate a combined index of β based on the single-scale index from 2004 to 2023. Furthermore, the X11 process was employed to identify the structural components of the time series of various types of hepatitis.

**Results:**

In the past 20 years, the proportion of patients with viral hepatitis in Zhejiang Province has changed significantly, and hepatitis B remains the main type of hepatitis, accounting for approximately 70% of all hepatitis cases. The proportion of hepatitis C and E cases has been increasing, whereas the proportion of hepatitis A cases has been decreasing since 2004 and has remained at a low level (approximately 3%) since 2010. The combined scaling exponents of hepatitis A, hepatitis B, hepatitis C, hepatitis E and unclassified hepatitis based on the random effects model were 0.88 (95% confidence interval(CI): 0.78 to 0.98), 0.78 (95% CI: 0.70 to 0.86), 1.18 (95% CI: 1.11 to 1.26), 0.91 (95% CI: 0.86 to 0.97) and 0.89 (95% CI: 0.79 to 1.00), respectively.

**Conclusion:**

In the past 20 years, the epidemic situation of hepatitis A, hepatitis B and unclassified hepatitis has shown a significant downward trend, whereas the proportions of hepatitis C and hepatitis E among those with viral hepatitis have increased annually. The combined scaling exponent and development trends of the five types of hepatitis show significant heterogeneity. Overall, hepatitis C exhibits superlinear characteristics, whereas other types of hepatitis exhibit sublinear characteristics. Different types of hepatitis exhibit distinct epidemic characteristics and require targeted prevention and control measures.

## Background

Viral hepatitis is an infectious disease that mainly leads to liver lesions caused by various hepatitis viruses. Hepatitis greatly increases the global disease burden and has become a major public health issue worldwide [1]. According to statistics, viral hepatitis causes 1.5 million deaths annually and seriously affects the quality of life of hundreds of millions of people [2]. Hepatitis A and hepatitis E are widely distributed intestinal infectious diseases worldwide and are classified as acute infections and self-healing [3]. In China, the incidence rate of hepatitis A was as high as 55.7 cases/100000 person-years in 1991 [4]. Hepatitis E is a substantial cause of illness and death worldwide, particularly among pregnant women [5]. Hepatitis B and C are bloodborne infectious diseases. In 2015, the global hepatitis virus caused 10 million new infections and 1.3 million deaths, of which 96% were caused by chronic infection caused by hepatitis B virus and hepatitis C virus [6]. Furthermore, hepatitis B virus and hepatitis C.

Viruses have parallel transmission routes, so a certain proportion of patients can have dual virus infection, which can lead to a 2–3-fold increased risk of advanced liver disease [6–9]. If treatment is delayed, chronic hepatitis can lead to life-threatening complications such as cirrhosis and hepatocellular carcinoma [10]. China is a high-risk area for viral hepatitis, which is also a serious public health problem.

In 2016, the World Health Organization set ambitious goals to eliminate viral hepatitis as a public health problem by 2030, including reducing new infections by 90% and reducing viral hepatitis deaths by 65% [2]. China is the country with the highest burden of viral hepatitis infection and will become a major contributor to the global elimination of viral hepatitis by 2030 [11]. In recent years, China has invested considerable manpower, funds, and security measures in controlling viral hepatitis. Since 2008, the country has included the hepatitis A vaccine in the national immunization plan. In 2015, all pregnant women were screened for HBsAg free of charge. In 2017, the National Food and Drug Administration approved direct antiviral drugs for hepatitis C to be launched in China. These prevention and control measures have changed the trend and epidemiological characteristics of viral hepatitis.

In recent years, time series analysis methods have been widely used in the analysis of infectious diseases. This study uses the X11 process in time series analysis to decompose the seasonal factors and long-term trends of various types of hepatitis to explore the trend changes in viral hepatitis in Zhejiang Province over the past 20 years. Moreover, the analysis method of complex urban systems provides a new perspective for understanding the characteristics of infectious diseases [12,13]. Many previous studies have shown that AIDS, influenza, dengue fever, COVID-19 and other infectious diseases have different scaling relationships with population size [14–17]. For example, a study using data from the United States, Brazil, Sweden and other countries found that the incidence of infectious diseases such as AIDS, influenza, dengue fever and the size of urban population were superlinearly scaled [14]. Another study showed a super linear relationship between confirmed COVID-19 cases and population size, indicating that the diagnosis rate is higher in large cities, while deaths did not show a super linear relationship with population size, indicating that the mortality rate in large cities is not higher than that in small and medium-sized cities [14]. Therefore, this study also employs a scaling law to explain the scaling relationship between hepatitis incidence and population size in Zhejiang Province, China. To provide scientific support for public health professionals and policy-makers to develop effective strategies to eliminate viral hepatitis.

## Materials and methods

### Setting and area of study

This is an ecological study on hepatitis in Zhejiang Province. Zhejiang Province is a provincial-level administrative region of the People’s Republic of China, with Hangzhou city as its capital. It is located on the southeast coast of China and spans 27°02′ ~ 31°11′ north latitude and 118°01′ ~ 123°10′ east longitude. There are 11 cities in Zhejiang Province. At the end of 2023, the permanent population of Zhejiang Province was approximately 65 million people.

### Data collection

Data on hepatitis patients diagnosed at medical institutions were collected between 01/01/2004 and 31/12/2023 from the China Disease Control and Prevention Information System(CDCPIS). The date on which we accessed data for research purposes from this system and conducted data organization is 26/09/2024. The population data at the county level (all counties in total) were updated by the company responsible for system operation and maintenance, and the new population data were imported into the system every December. The incidence rate of hepatitis was computed by the system and exported. The diagnosis of hepatitis was based on the ‘Diagnostic criteria for hepatitis’. The formula for calculating the incidence of acquired hepatitis in Zhejiang Province from 2004 to 2023 is the number of reported cases in the period divided by the total average population in the year. We can also access information that could identify individual participants during or after data collection.

### Ethics statement

This study was reviewed and approved by the Ethics Committee of the Zhejiang Provincial Center for Disease Control and Prevention. All the data of the individuals were kept confidential as requested and followed the Law of the Prevention and Treatment of Infectious Diseases in the People’s Republic of China. In accordance with the Law of the Prevention and Treatment of Infectious Diseases in the People’s Republic of China, the demographic and disease information of hepatitis patients must be reported. When clinical doctors diagnose infectious disease, they will inform the patient and their guardian of the diagnosis results and at the same time the physician fill out the infectious disease report card and enter it into CDCPIS. As only surveillance information was analyzed, this study did not involve human research and the information that can identify personal identity is not required for this study, informed consent was exempted by the Ethical Institutional Review Board.. All the methods employed in the study were performed in accordance with the applicable guidelines and regulations.

### Scaling law and meta-analysis [14]

The scaling law of an urban system reveals the scaling relationship between urban indicators and population size at the same time point, and its function form is a power function [18,19]:


$$Y=Y_{0}N^{\beta }$$ (1)

In Equation (1), *Y* represents the urban indicator (such as the cumulative number of confirmed cases of syphilis in county-level units as of a certain year). *N* is the population size; *Y*_*0*_ and β are parameters, where β is the scaling exponent. By taking both sides of Equation (1) with a base logarithm of 10, we can obtain Equation (2):


$$\mathrm{lg}Y=\beta \times \mathrm{lg}N+\mathrm{lg}Y_{0}$$ (2)

Equation (2) is a linear function. After the logarithms of the confirmed cases and population size are obtained, a linear function is used for fitting, and the slope β of the fitted line is the scaling exponent. Despite the availability of other nonlinear fitting methods, linear fitting is widely used in scaling law fitting because of its simplicity and ease of operation [20,21]. According to the relationship between *β* and 1, the urban indicators can be divided into three types: ① The indicator exhibits a superlinear scale relationship with population size (β> 1), and the scaling exponent of this type is approximately 1.15. ② The indicator has a sublinear scale relationship with population size (β< 1), and the scaling exponent is approximately 0.85. ③ The scale is linearly proportional to the population size, and the scaling exponent ‘β’ is usually approximately equal to 1.

Fixed-effects and random-effects meta-analyses were used to calculate a combined index of β based on the single-scale index from 2004 to 2023. Inverse variance weighting was used for pooling. The heterogeneity of the scaling exponent in different years was tested to select the fixed effects model or random effects model [22]. The scaling law analysis and meta-analysis of the scale index were performed in R Studio (version 1.2.5001). The *I*^*2*^ statistic is an indicator of heterogeneity. The larger the value is, the stronger the heterogeneity. Moreover, a value greater than 75% indicates high heterogeneity. A *p* value less than 0.05 represented statistical significance for heterogeneity of the scale index in different years, and the random effect result was applied.

### X11 process

The X-11 process is a time series seasonal adjustment process compiled by the United States Bureau of Investigation. The X-11 process is based on the assumption that any time series can be decomposed into long-term trend fluctuations $T_{\text{t}}$, seasonal fluctuations $S_{t}$, trading days $D_{t}$ and irregular fluctuations $I_{t}$. This study uses monthly incidence data for analysis; therefore, trading day factors are not considered. The time series in this study can be multiplied and decomposed as follows:


$$x_{t}=T_{t}S_{t}I_{t}$$ (3)

The X-11 process involves extracting seasonal and random influences from the original sequence to obtain the most accurate long-term trend patterns. The moving average method is commonly used in the calculation of this process. Multiple short-term centralization moving averages are used to eliminate random fluctuations, and periodic moving averages are used to eliminate trends. A total of 11 moving averages are required throughout the process, hence the name X-11 process. The methods above were computed via SAS 9.2 (SAS Institute Inc., Cary, NC).

## Results

### Basic epidemiological characteristics

From 2004 to 2023, a total of 20,457 cases of hepatitis A, 464,726 cases of hepatitis B, 47,589 cases of hepatitis C, 40,599 cases of hepatitis E, and 51,758 cases of unclassified hepatitis were reported in Zhejiang Province. The total number of deaths corresponding to each type of viral hepatitis was 2, 160, 31, 22, and 30, respectively, with the majority of deaths being caused by hepatitis B (65.31%). However, in terms of the crude mortality rate, hepatitis C had the highest mortality rate (0.065%), followed by unclassified hepatitis (0.058%), hepatitis E (0.054%), hepatitis B (0.034%), and hepatitis A (0.0010%). Among each type of viral hepatitis, male cases are predominant, with corresponding male to female case ratios of 1.74, 2.27, 1.62, 2.07, and 1.68, respectively. Similarly, the majority of deaths were male, with a total of 186 reported male deaths and only 59 female deaths.

In terms of age distribution, the majority of hepatitis A patients were in the 30–49 years age group, accounting for 41.21% of all hepatitis A patients. The majority of hepatitis B patients were in the 20–39 years age group, accounting for 47.32% of all hepatitis B patients. The majority of hepatitis C patients were in the 30–49 years age group, accounting for 53.30% of all hepatitis C patients. The majority of hepatitis E patients were in the 40–59 years age group, accounting for 45.92% of all hepatitis E patients. The majority of patients with unclassified hepatitis were in the 30–49 years age group, accounting for 40.73% of all patients. From the perspective of death cases, 98.78% of the deaths occurred in the population aged 20 years and above, with the highest proportion of deaths occurring in the 40-59 years age group (47.35%).

Farmers constitute the population with the highest incidence of each type of hepatitis, accounting for 39.37%, 42.59%, 31.14%, 44.69%, and 43.02%, respectively. Except for hepatitis C, the second largest group of people with other types of hepatitis are workers, including 12.23% with hepatitis A, 15.24% with hepatitis B, 12.24% with hepatitis E, and 13.82% with unclassified hepatitis. The second largest proportion of people with hepatitis C is household and unemployed individuals (16.10%).

### Trend of hepatitis

**Fig 1:**
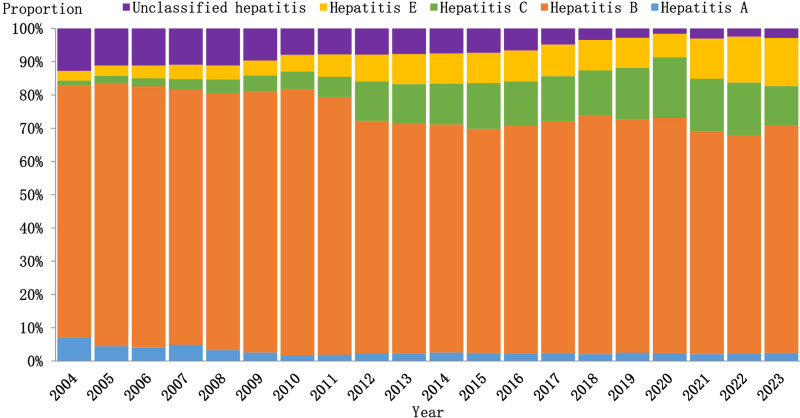
Trends in the proportions of various types of viral hepatitis in Zhejiang Province from 2004 to 2023.

From 2004 to 2023, the proportion of patients with viral hepatitis in Zhejiang Province changed significantly, and hepatitis B remained the main type of hepatitis, accounting for approximately 70% of all hepatitis cases. In the past 20 years, the proportion of patients with hepatitis C and E has been increasing, whereas the proportion of patients with hepatitis A has been decreasing since 2004 and has remained at a low level (approximately 3%) since 2010. In addition, the proportion of unclassified hepatitis cases showed a significant downward trend (Fig 1).

**Fig 2:**
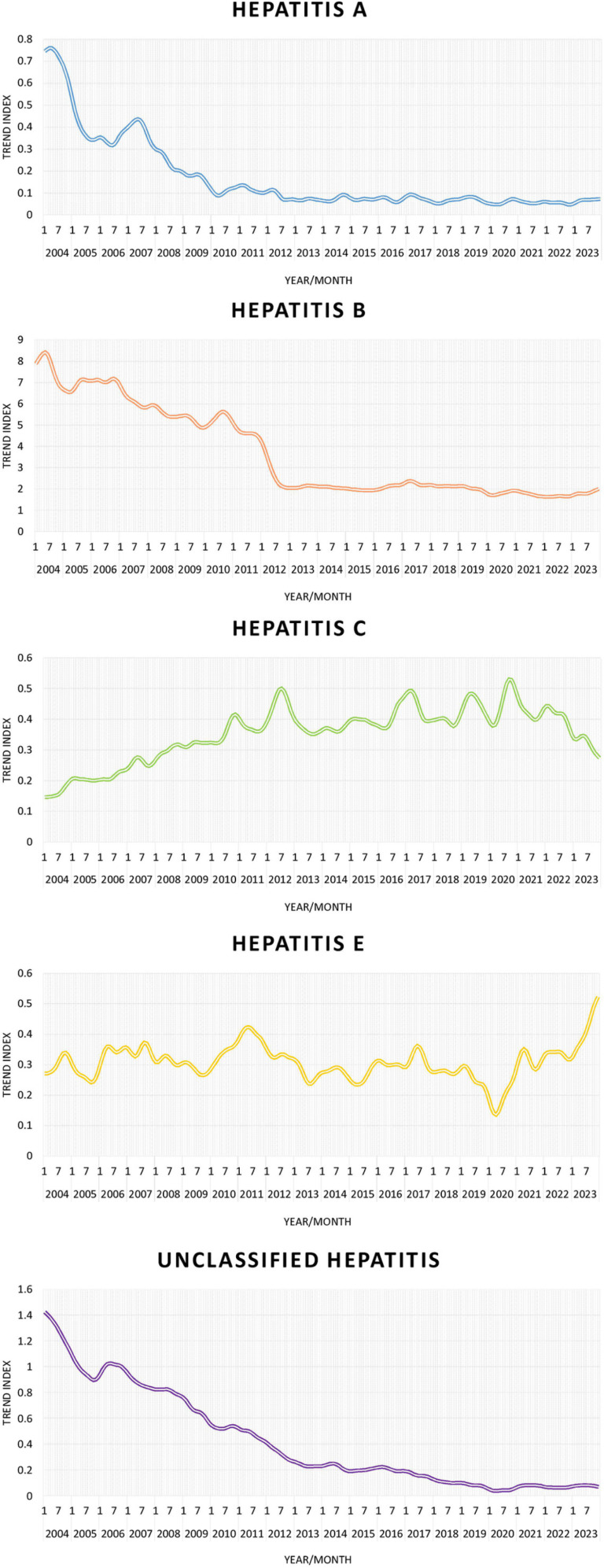
Trend components extracted from the X11 process for various types of viral hepatitis in Zhejiang Province from 2004 to 2023.

From the perspective of trend components, the hepatitis A epidemic has shown an overall downward trend since 2004. A slight rebound was noted in 2007, but a significant decrease has occurred since 2008. This trend has remained since then with a low level of fluctuation. The trend of the hepatitis B epidemic can be roughly divided into two stages. First, before 2012, the epidemic showed a continuous downward trend, followed by a sudden sharp decline in 2012. Second, after 2012, it showed a stable development trend. The hepatitis C epidemic showed an overall trend of first increasing and then decreasing. Before 2012, the epidemic continued to increase and then fluctuated at a relatively stable level from 2012 to 2020. After 2021, the overall epidemic showed a downward trend. Unlike other types of hepatitis, the hepatitis E epidemic has not shown a significant downward trend, with only one sudden decline in 2020. Thereafter, the epidemic has shown a clear upward trend. From 2004–2019, the epidemic of unclassified hepatitis showed a significant continuous declining trend. Since then, the epidemic has stabilized at a relatively low level (Fig 2).

**Fig 3:**
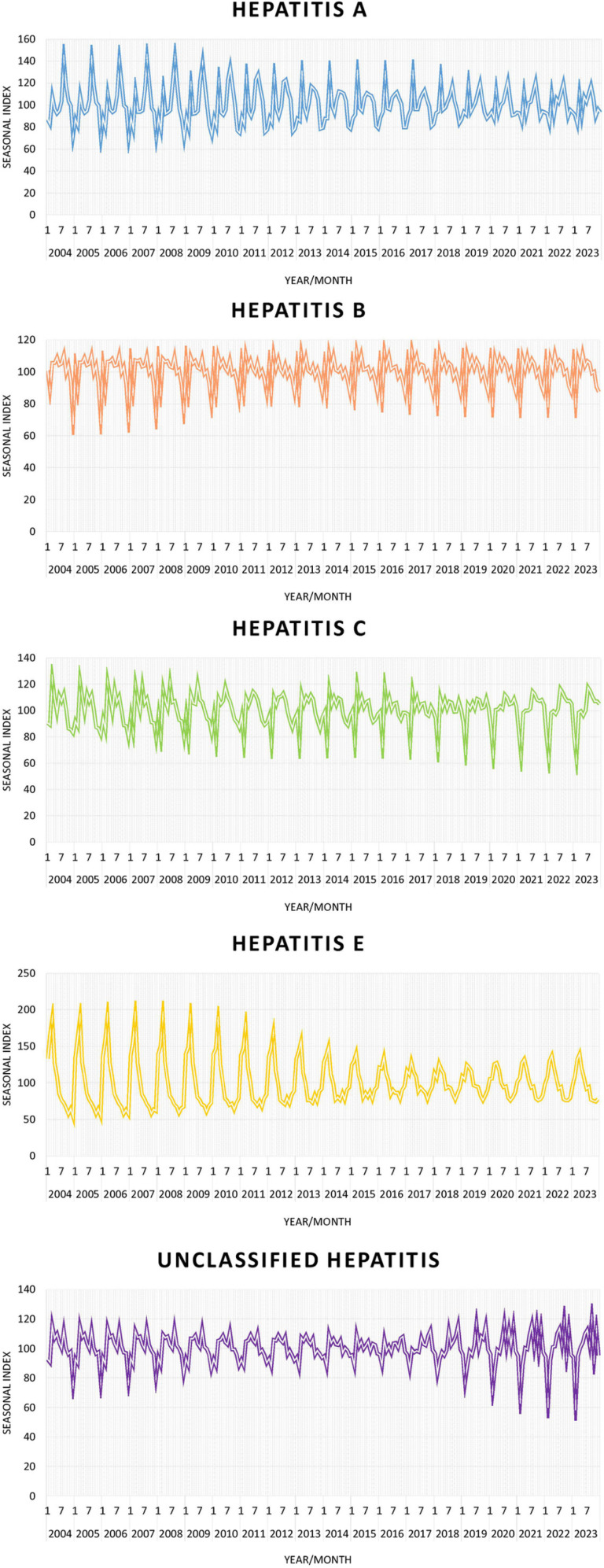
Seasonal components extracted from the X11 process of various types of viral hepatitis in Zhejiang Province from 2004 to 2023.

From a seasonal perspective, hepatitis A experiences two peak epidemics each year, one in July and August and the other in March. Interestingly, the component values of the summer peak have gradually decreased. Before 2011, the summer peak was greater than the spring peak; however, between 2011 and 2019, the spring peak exceeded the summer peak. Since then, the two peaks have remained relatively consistent. The epidemic situation of hepatitis B did not show a significant seasonal peak, and its seasonal factor composition remained relatively stable from March to October. However, there were two valleys in February and December each year. Like those of hepatitis B, the seasonal factor components of the hepatitis C epidemic are relatively high from March to September, and a clear trough is observed in February. The seasonal factor components of the hepatitis E epidemic clearly exhibit a single peak within the year, which occurs between February and April. Another obvious feature is that the seasonal factor peak of hepatitis E has gradually decreased since 2011. The trend chart of seasonal factors in the epidemic of unclassified hepatitis shows a certain degree of similarity with the manifestations of hepatitis B and C. Overall, it shows a relatively stable trend from March to September and a relatively low trend in February and December (Fig 3).

### Scaling law of hepatitis

**Fig 4:**
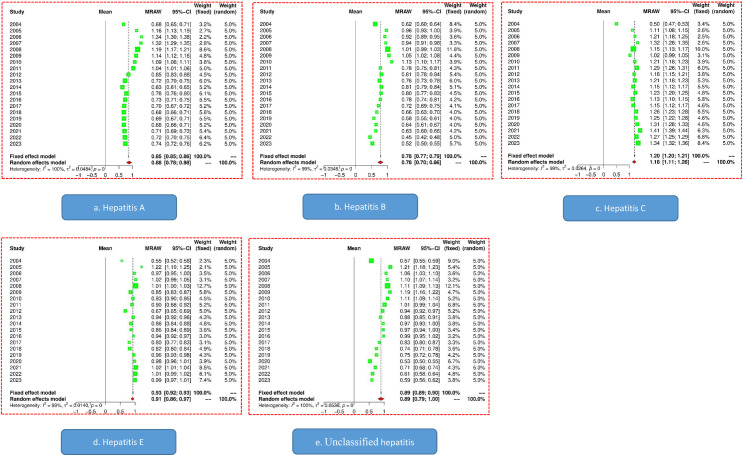
Forest plot of the scaling exponent of hepatitis incidence from 2004 to 2023 (a. forest plot of hepatitis A; b. forest plot of hepatitis B; c. forest plot of hepatitis C; d. forest plot of hepatitis E; e. forest plot of unclassified hepatitis).

From 2005 to 2011, the scaling exponent of hepatitis A incidence showed a downward trend. Since 2012, the scaling exponent of hepatitis A has been less than 1. The Higgins *I*^*2*^ value of 100% (*p* < 0.001) indicated heterogeneity in the scaling exponents of each year. Therefore, the results of the random effects model were used; that is, the combined scaling exponent was 0.88 (95% CI: 0.78 to 0.98) (Fig 4a).

For hepatitis B, except for the years 2008 to 2010, the scaling exponent for all other years was less than 1. The Higgins *I*^*2*^ value of 99% (*p* < 0.001) indicated heterogeneity in the scaling exponents of each year. Therefore, the results of the random effects model were used; that is, the combined scaling exponent was 0.78 (95% CI: 0.70 to 0.86) (Fig 4b).

Unlike other types of hepatitis, the scaling exponent of hepatitis C in each year is greater than 1, and the index in 2004 may be an outlier. The Higgins *I*^*2*^ value of 99% (*p* < 0.001) indicated heterogeneity in the scaling exponents of each year. Therefore, the results of the random effects model were used; that is, the combined scaling exponent was 1.18 (95% CI: 1.11 to 1.26) (Fig 4c).

In most years, the scaling exponent of hepatitis E is less than 1. However, in the past five years, its scaling index has tended to approach 1. The Higgins *I*^*2*^ value of 99% (*p* < 0.001) indicated heterogeneity in the scaling exponents of each year. Therefore, the results of the random effects model were used; that is, the combined scaling exponent was 0.91 (95% CI: 0.86 to 0.97) (Fig 4d).

A high degree of similarity was noted between the scaling exponent trends of unclassified hepatitis and hepatitis A. When 2011 was used as the dividing point, the scale exponent before 2011 was greater than 1; however, afterward, it was less than 1. The Higgins *I*^*2*^ value of 99% (*p* < 0.001) indicated heterogeneity in the scaling exponents of each year. Therefore, the results of the random effects model were used; that is, the combined scaling exponent was 0.89 (95% CI: 0.79 to 1.00) (Fig 4e).

## Discussion

Among the five types of hepatitis, hepatitis B accounts for 74% of the total number of cases and 65% of the total number of deaths, making it the hepatitis with the highest number of infections and deaths. This result is consistent with previous literature reports [23]. Previous studies have shown that an estimated 291 million people worldwide suffer from chronic hepatitis B virus (HBV) infection. Moreover, HBV is highly prevalent in China, and the high incidence of HBV in history has led to the formation of a reservoir of approximately 86 million chronic infections, accounting for 30% of the global burden of chronic HBV infection [24]. However, interestingly, hepatitis C has the highest mortality rate, and the mortality rates of unclassified hepatitis and hepatitis E are also similar to those of hepatitis C. The mortality rate of hepatitis B is lower than those of the first three types of hepatitis. For hepatitis C, 22 of the 31 deaths were reported in the past two years, which may be related to China’s goal of eliminating hepatitis C by 2030, as standardized reporting of hepatitis C deaths has strengthened in recent years [25]. The reason for the high mortality rate of hepatitis E may be that some cases are prone to progression to fulminant hepatitis. The deaths of patients with unclassified hepatitis mainly occurred before 2012, accounting for 90%, which may be related to the failure to clarify the cause of the cases in a timely manner and delayed treatment. The five types of hepatitis are mostly noted in male cases, which may be related to more risk exposure factors for men, such as a higher risk of an unclean diet and more unsafe use of blood products, needles, knives, etc. The demographic distribution characteristics of the cases indicate that the majority of cases are among farmers and workers aged 30–49 years, which may also be related to the high risk exposure factors mentioned above.

The hepatitis A epidemic has shown a significant downward trend since 2004, especially after 2010, when the overall epidemic remained at a low level, which is consistent with previous research results [3,23]. Since 2008, Zhejiang Province has included hepatitis A vaccines in the childhood immunization plans of five cities and achieved full vaccine coverage by 2010. Moreover, at the national level, the number of hepatitis A vaccine batches issued is far greater than the number of births per year, and the vaccination rate is high, which has significantly reduced the reported incidence rate of vaccine coverage and other age groups [26]. This study revealed that the decline in the hepatitis A epidemic in Zhejiang Province may be related to socioeconomic development, improvements in the living environment and hygiene conditions, and increased public self-protection awareness. However, the introduction of vaccines remains the most important factor [4,27,28]. Since 1992, Zhejiang Province has promoted the vaccination of newborn hepatitis B vaccines throughout the province, which has led to a steady decline in the incidence rate of hepatitis B. Furthermore, from 2007 to 2010, Zhejiang Province organized leak detection and supplementary vaccination of the hepatitis B vaccine for 1,088,900 children under the age of 15, and the vaccination completion rate reached more than 95%. This work may play an important role in significantly reducing the incidence of hepatitis B after 2012. On the other hand, relevant research shows that only 35% of the cases reported as acute hepatitis B meet the diagnostic criteria, and chronic hepatitis B also has many problems with repeated reporting over the years [29,30]. In response to these problems, Zhejiang Province further standardized the diagnosis and reporting of hepatitis B in 2012, thus reducing false reports and repeated reports and leading to a rapid decline in the reported incidence level. The increasing trend of hepatitis C may be related to the following factors: First, the public lacks understanding and attention to transmission routes and prevention of hepatitis C. Second, although blood product testing has become increasingly sophisticated and strict in recent years, there is a window for anti-HCV antibody testing, and a small number of cases may not produce anti-HCV antibodies after infection, which affects the screening of blood and blood products and leads to infections related to blood transfusion and hemodialysis. Third, iatrogenic infection, such as dental treatment and endoscopy, and operation infections, such as those encountered during tattoo, beauty, acupuncture and moxibustion procedures, contribute to increasing trends. Fourth, social behavioral factors, such as drug users sharing injection needles, sexual contact transmission, etc., also contribute [23]. However, since 2015, China has started screening blood donors who are negative for HCV antibodies for HCV RNA, and the recruitment of blood donors has shifted from paid to voluntary. In addition, disinfection standards, hospital infection control standards, and hepatitis C prevention and control guidelines have been developed and implemented [25]. The implementation of these measures may have promoted a decrease in the incidence level after 2020. Unlike other types of hepatitis, this study did not find a decreasing trend in the incidence of hepatitis E, which may be related to multiple factors. First, despite the use of the hepatitis E vaccine, its usage is still low, and the population coverage is limited. Second, the current prevalence of hepatitis E virus in China has shifted from type 1 to type 4, indicating a shift in the transmission mode of hepatitis E from fecal oral transmission to zoonotic transmission. This also explains why the incidence of hepatitis E has not decreased synchronously in the context of a general decline in the incidence of most intestinal infectious diseases [4]. In 2020, the incidence of hepatitis E reached a low point, which may be related to the reduction in population activity during the emergency response to the COVID-19 epidemic [31]. In addition, the increase in incidence in recent years may be related to improvements in monitoring sensitivity. Since 2004, the incidence of unclassified hepatitis has continuously declined, mainly due to improvements in diagnostic technology, which has enabled an increasing number of cases of viral hepatitis to be clearly classified.

There are two peaks in the incidence of hepatitis A, one occurring from July to August, which is consistent with the high incidence of intestinal infectious diseases in summer and may be related to the listing of seafood products. The other effect occurring in March may be related to the “Spring Festival effect”, as large-scale population movements and increased family gatherings facilitate the spread of pathogens between infectious sources and susceptible populations. Moreover, population susceptibility increases due to fatigue, leading to an increase in incidence after the Spring Festival in February. However, from the trend of changes in the two seasonal factors, the summer factor decreased, which may be related to the improvement in the food hygiene level. In contrast, the spring factor increased, indicating the need to strengthen healthy diet education during the Spring Festival. Hepatitis B, hepatitis C and unclassified hepatitis do not show obvious seasonal characteristics, which is consistent with the epidemiological trends of these diseases. In addition, the low incidence from December to February of the following year may be related to the reduction in the number of cases seeking medical attention due to traditional holidays during this period. The seasonal factor of hepatitis E is very obvious, showing a single peak characteristic from February to April, which is similar to the Spring Festival holiday effect noted for hepatitis A. In addition, pigs infected with HEV are also an important source of hepatitis E virus transmission. The low temperature in winter can increase the survival time of HEV in pigs, thereby increasing the possibility of exposure to infection. In addition, the seasonal factors of hepatitis E show a decreasing trend, which may indicate that, owing to changes in dominant subtypes and transmission modes, transmission sources are more widespread and that transmission routes are more complex in zoonotic transmission modes, increasing the difficulty of prevention and control.

The relationship between the hepatitis A epidemic and population size has undergone a transition from a superlinear correlation to a sublinear correlation. Before the promotion of the hepatitis A vaccine, the hepatitis A epidemic was more prominent in urban areas with larger populations, as the growth rate of the epidemic exceeded the growth rate of the population. A recent study revealed that the positivity rate of hepatitis A virus in shellfish in coastal areas is 4%, which may have been relatively high in the past and potentially caused by outbreaks of food such as clams [32]. Therefore, before the use of vaccines, large cities had a higher incidence of epidemics due to the denser population and greater consumption of seafood. After the use of vaccines, the growth of the epidemic in urban areas with larger populations was actually lower than that in areas with smaller populations. This suggests that vaccination coverage in urban areas with larger populations may be greater, resulting in better epidemic control effects. In contrast, small cities or rural areas may have insufficient vaccination coverage.

In the past 20 years, the epidemic situation of hepatitis B has shown a sublinear relationship with overall population size, indicating that the growth rate of the epidemic of hepatitis B has been faster in cities or rural areas with smaller populations than in large cities. According to the literature, chronic hepatitis B is currently the main type of hepatitis B in China, and the proportion of acute hepatitis B cases is less than 20% [30]. As the hepatitis B vaccine has been included in the planned immunization program, the epidemic of hepatitis B has declined significantly. In this context, the relatively rapid increase in the incidence of the hepatitis B epidemic in small cities or rural areas may be related to the misdiagnosis of hepatitis B and the repeated reports of chronic cases, which lead to the false high incidence of hepatitis B in these areas.

The relationship between the hepatitis C epidemic and the size of the urban population is highly linear, indicating that the increase in the incidence of the epidemic in large cities is faster than that in small cities or rural areas. Related research shows that the main high-risk population of hepatitis C patients has gradually become patients who are receiving drugs, patients who are receiving hemodialysis, patients who are coinfected with human immunodeficiency virus (HIV), or patients who are infected with the hepatitis B virus [25,33]. At present, the use of injected drugs and high-risk sexual behavior has become the main route of HCV transmission in China [25]. Therefore, in large cities with larger populations, due to frequent population movements and faster economic development, there may be more cases of drug abuse and high-risk sexual behavior, leading to a superlinear relationship with hepatitis C.

The hepatitis E epidemic shows a sublinear correlation with the overall population size. This result suggests that in small cities or rural areas, there may be a greater risk of infection caused by unclean drinking water or diet due to poor sanitation conditions. Another noteworthy point is that in recent years, the scaling exponent of the hepatitis E epidemic has begun to approach a linear pattern. This finding indicates that as the population size increases, the hepatitis E epidemic also increases in a linear fashion. This may be related to the shift in the dominant subtypes and transmission patterns of hepatitis E, while also indicating a high degree of similarity in the infection risks faced by populations in cities of different sizes.

The scaling exponent of unclassified hepatitis also transitions from a superlinear mode to a sublinear mode. Because unclassified hepatitis cannot be clearly diagnosed as a specific type of hepatitis, the reported incidence level of this type of hepatitis is closely related to local diagnostic capabilities. In cases where the overall diagnostic level is not high in the early stage, more cases may be reported in cities with larger population sizes. With improvements in the diagnostic level, the accuracy of diagnosis in large cities may be significantly improved, increasing the likelihood of clarifying the specific subtype of hepatitis. However, in small cities or rural areas where diagnostic technology is relatively backward, a relatively high proportion of hepatitis cases cannot be clearly classified.

Several limitations should be noted in our study. First, in the reported cases, erroneous reports caused by misdiagnosis and repeated reports of chronic cases across years were not excluded, which may have caused a certain bias in the accuracy of the analysis results. Second, there are still some uncertainties about the scaling law. One concern is the relationship between the scaling exponents of urban indicators and population size in different urban areas. Another concern is the discrepancy between the scaling factor obtained from linear fitting and the power function index obtained from nonlinear fitting. Third, owing to insufficient data, the influence of covariates on the scaling law has not been corrected, which needs to be further improved and verified in future research. Fourth, owing to the low incidence of hepatitis D, this virus was not included in the analysis. Fifth, the data for this study come from passive monitoring systems, and the characteristics of untreated cases cannot be determined. Therefore, there may be some bias in the epidemiological characteristics presented in the reported data.

## Conclusion

In the past 20 years, the epidemics of hepatitis A, hepatitis B and unclassified hepatitis have shown a significant downward trend, while the proportions of hepatitis C and hepatitis E among those with viral hepatitis have increased every year. Both the hepatitis A and hepatitis E epidemics have strong seasonal factors, with both experiencing seasonal peaks in February and March. In addition, hepatitis A also exhibits summer peak characteristics. The combined scaling exponent and development trends of the five types of hepatitis show significant heterogeneity. Overall, hepatitis C exhibits superlinear characteristics, whereas other types of hepatitis exhibit sublinear characteristics. Different types of hepatitis exhibit distinct epidemic characteristics and require targeted prevention and control measures. For hepatitis A and hepatitis B, maintaining a high vaccination rate is the key to preventing and controlling the epidemic, and special attention should be given to hepatitis A vaccination in small cities and rural areas. For hepatitis C, the monitoring system should be improved to identify high-risk populations and develop precise prevention and control measures. For hepatitis E, in addition to strengthening health education, it is necessary to further understand the use of the hepatitis E vaccine, evaluate the impact of vaccine use on the incidence of the disease in the population, and promote the use of the hepatitis E vaccine in the population. In addition, we should further standardize the diagnosis of chronic hepatitis B and unclassified hepatitis, avoid misdiagnosis and repeated reports, and improve the quality of the reported data. In future study, we will attempt to apply spatial feature data of the diseases and conduct spatiotemporal analysis by combining it with temporal feature data, such as spatiotemporal clustering detection and spatiotemporal multi-component analysis. At the same time, we will collect more data on influencing factors and explore the main factors driving the development of different types of hepatitis epidemics.

## Supporting information

S1 File Database of the incidence rates of various types of viral hepatitis in Zhejiang Province from 2004 to 2023. (XLSX)
